# Accumulation of “Old Proteins” and the Critical Need for MS‐based Protein Turnover Measurements in Aging and Longevity

**DOI:** 10.1002/pmic.201800403

**Published:** 2019-09-10

**Authors:** Nathan Basisty, Anja Holtz, Birgit Schilling

**Affiliations:** ^1^ The Buck Institute for Research on Aging Novato CA USA

**Keywords:** aging, long‐lived proteins, mass spectrometry, protein turnover, proteostasis

## Abstract

Aging and age‐related diseases are accompanied by proteome remodeling and progressive declines in cellular machinery required to maintain protein homeostasis (proteostasis), such as autophagy, ubiquitin‐mediated degradation, and protein synthesis. While many studies have focused on capturing changes in proteostasis, the identification of proteins that evade these cellular processes has recently emerged as an approach to studying the aging proteome. With advances in proteomic technology, it is possible to monitor protein half‐lives and protein turnover at the level of individual proteins in vivo. For large‐scale studies, these technologies typically include the use of stable isotope labeling coupled with MS and comprehensive assessment of protein turnover rates. Protein turnover studies have revealed groups of highly relevant long‐lived proteins (LLPs), such as the nuclear pore complexes, extracellular matrix proteins, and protein aggregates. Here, the role of LLPs during aging and age‐related diseases and the methods used to identify and quantify their changes are reviewed. The methods available to conduct studies of protein turnover, used in combination with traditional proteomic methods, will enable the field to perform studies in a systems biology context, as changes in proteostasis may not be revealed in studies that solely measure differential protein abundances.

## 1 Introduction

The maintenance of protein homeostasis (proteostasis)^1^ in cells is crucial for normal cellular function, physiological protein folding, and for cellular proteome stability and functionality. Dysregulation in proteostasis can lead to severe problems, such as protein damage, misfolding, and aggregation, disruption of the proteostasis network (PN), and ultimately may contribute to aging and age‐related diseases. The PN is a multi‐compartmental system that coordinates protein synthesis, folding, disaggregation, and degradation, and is composed of translational machinery, molecular chaperones and co‐chaperones, the ubiquitin–proteasome system, and autophagy machinery.^2^ During aging and related diseases, disruption in all components of the PN has been widely documented. In more recent years, mass spectrometric (MS) methods have been developed to enable comprehensive measurements of protein turnover directly at the level of individual proteins in vivo. Using a combination of stable‐isotope labeling, MS, and specialized software analysis, has allowed insight into dynamic alteration of the proteome^3^, ^4^ and the potential loss of protein enzymatic function. Here, we review how proteome‐wide measurements of protein turnover have been applied in the context of aging and longevity. We believe this is a quickly growing field in proteomics research as protein turnover plays such a key regulatory role in cells. To assess changes in proteostasis during aging, protein turnover measurements can complement other proteomics datasets, for example, more typical protein expression changes. Moving toward systems biology investigations of aging integrating protein turnover workflows will be highly relevant and can provide more system‐relevant results. Combining stable‐isotope labeling with MS clearly provides tools that are not available with other more classical “bulk” measurements that can only determine global changes. Proteomics technologies, however, provide granularity for protein turnover changes on the individual protein level, and can thus provide relevant changes of specific proteins, that then might be used as specific marker protein or biomarker for the specific conditions measured. While global protein turnover changes are clearly interesting, it is important to know i) which proteins may show a particularly large change in protein turnover (slowing or accelerating), ii) which proteins may behave against the general trend, iii) are there certain groups of proteins that behave similarly in their protein turnover changes over time that belong to a common pathway, and iv) are there certain proteins for which protein turnover stays relatively constant during aging; MS technologies can clearly provide these detailed results. We also emphasize how these technologies have allowed us to discover groups of particularly long‐lived proteins that persist for years in many organisms, and their role during aging.

## 2 Long‐Lived Proteins and Aging

Advancing capabilities to measure individual protein turnover in a comprehensive manner, rather than only measuring bulk changes in protein synthesis/degradation rates, have enabled the identification of proteins that are particularly long‐lived. Such long‐lived proteins (LLPs) have been found in several different subcellular compartments and include nuclear pore complexes, histones, structural proteins of the extracellular matrix (ECM), myelin sheath proteins, or eye lens crystallins.^5^, ^6^ LLPs, due to very low rates of turnover, are inherently at an increased risk for accumulation of potentially damaging posttranslational modifications (PTMs), such as oxidation, which may become particularly relevant during cellular aging. For example, the very long‐lived nuclear pore complex proteins, such as the scaffold nucleoporins Nup107/160, remain incorporated in the nuclear membrane during the entire lifespan of a cell and have been shown to deteriorate with age, causing a loss of nuclear integrity in post‐mitotic cells.^6^ Protein turnover measurements have revealed the complex role of extremely long‐lived nuclear pore proteins in the rat brain,^7^ and their role in providing stability of essential cellular structures.^8^ To identify LLPs, the authors performed ^15^N stable‐isotope pulse‐chase labeling of rats, and subsequently quantitative mass spectrometric methods were used to assess brain and liver tissues. In neurons, extreme LLPs were observed that showed lifespans of months or even years.^7^, ^8^

It has been recognized that the presence of LLPs is often accompanied by gradual degradation of the proteins or damage and modifications to specific amino acids within proteins.^9^ Proteins of the eye, particularly lens α‐crystallins, can undergo racemization events affecting aspartate, asparagine, and serine residues, while asparagine and glutamine often undergo deamidation with age.^10^ Additional reports describe changes in proteostasis and PTMs of lens proteins, as well as crystallin aggregation occurring in the lens during aging.^11^ Modifications and changes to LLPs may be more common than anticipated and potential progression during age‐related human diseases may lead to deterioration and loss of function of LLPs.

## 3 Protein Aggregation, Modifications, and Damage of Aged Proteins

Changes in protein turnover and proteostasis have been directly linked to aging and age‐related diseases—accelerated or slowed protein turnover can affect enzymatic function, protein recycling, protein–protein interactions, and other processes. It is interesting to hypothesize whether aged proteins contain more PTMs that may result in protein damage or lead to protein aggregation. Several recent studies have combined mass spectrometric protein turnover studies with simultaneous assessment of the corresponding protein PTM profile. For example, Villen et al. recently measured protein turnover rates for 3160 proteins in exponentially growing yeast and determined that protein localization, protein complex formation, and connectivity greatly influence protein half‐lives.^12^ Assessing PTM profiles, they found that proteins with faster turnover showed higher ubiquitination site occupancy while occupancy for other PTMs was similar between proteins with short or longer half‐lives.^12^ In addition, Basisty et al. recently investigated age‐related increases in accumulation of long‐lived aggregating proteins and correlated their ubiquitination status and insolubility.^13^ They also reported that age‐related decline in proteostasis led to accumulation of ubiquitinated proteins in aggregates, and that a large proportion of ubiquitinated proteins were not turned over in aged animals.^13^

The eye in particular contains abundant levels of the very long‐lived protein crystallin.^14^ Crystallin exhibits extremely slow protein turnover or fails to turnover altogether, and is thus particularly prone to accumulation of damage.^15^ In addition, ECM proteins such as collagen and elastin are also very slowly turned over and extremely long‐lived.^15^ As protein damage accumulates with age, the physical properties of the ECM exhibit increased rigidity and resistance to denaturation.^16^ The decline in proteostasis with aging and the resulting changes of protein half‐lives, PTM status, and propensity for aggregation appear to be interconnected, however, more studies are needed to fully understand these complex mechanisms.

## 4 Protein Turnover Measurements using Comprehensive Proteomics Workflows

Measurement of in vivo protein turnover, synthesis, and degradation in aging and age‐related diseases has typically been performed in well‐defined model organisms in aging research such as *S. cerevisiae*, *C. elegans*, *D. melanogaster*, and *M. musculus*.^17^ Numerous studies have utilized stable isotope labeling approaches to calculate in vivo protein turnover rates in humans as well, particularly in the context of muscle protein turnover,^18^, ^19^, ^20^, ^21^, ^22^ although a few others have examined LLPs.^23^ Here, we will briefly review the experimental design, methods, and analysis tools available to perform such investigations and highlight recent methodological advances with an emphasis on the identification of LLPs. For a more comprehensive review of the methodological details applied to studies of protein turnover in aging and longevity, we refer readers to other reviews.^17^, ^24^, ^25^, ^26^

MS‐based proteomic workflows coupled with metabolic (stable isotope) labeling are most often applied for the calculation of protein turnover rates or the identification of LLPs. However, other methods such as live‐imaging of fluorescently labeled proteins have also been successfully employed.^27^ MS approaches hold a clear advantage due to minimal interference on protein functions and cellular processes by the incorporation of stable isotopes. MS‐based approaches also offer superior throughput and accuracy, as well as simpler experimental design and preparation. Due to these benefits, MS‐based proteomics are the most commonly chosen experimental approach. Importantly, modern MS‐based strategies are capable of identifying and directly measuring turnover of thousands of proteins per sample with essentially the same preparation and protein material requirements as strategies focusing on only a handful of proteins. As opposed to the use of bulk proteome turnover measurements or relying on markers of proteostatic processes (i.e., autophagy, proteasome activity, etc.) to infer changes in turnover of a protein of interest, the turnover of each protein can be measured directly using modern MS approaches.

**Figure 1:**
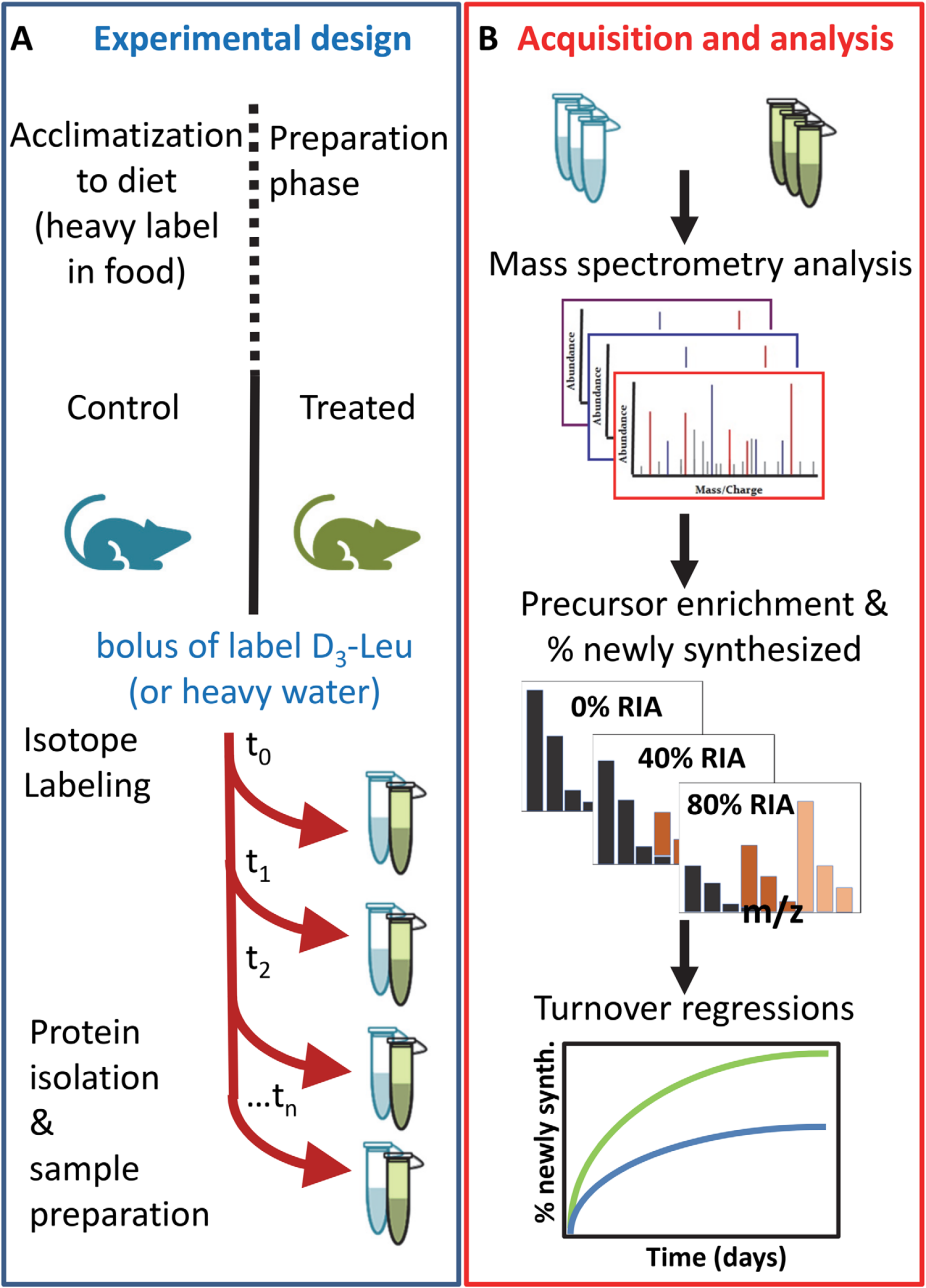
Protein turnover workflow. A) The use of heavy‐labeled amino acids generally requires a synthetic diet of a similar composition to regular chow, and it is important to acclimate animals to the non‐labeled synthetic diet for a few weeks prior to the start of the experiment. Mouse treatments, if used, are usually administered prior to supplementation of heavy label. The stable isotope label is typically supplemented in the chow. Tissues from all treatment groups are then collected at several time points, usually on the order of days to weeks, and processed for mass spectrometry analysis. B) For comprehensive survey of protein turnover, samples are analyzed by mass spectrometry using data‐dependent acquisition. An analysis of peptide isotopomer peaks is then conducted using specialized software (e.g., Topograph) to determine the enrichment of label in the precursor pool and the percentage of each protein that is newly synthesized. For each protein, a regression of the fraction that is newly synthesized is then performed to determine its rate of turnover. Figure 1 was adapted and modified based on a schematic of a recent publication under the terms and conditions of the Creative Commons Attribution License 4.0.^24^

For MS‐based determination of protein turnover rates, cells or organisms under investigation are metabolically labeled by supplementing a synthetic, stable‐isotope‐enriched diet. In mammals, the label of choice is usually heavy isotopes of amino acids, such as [^13^C_6_]‐arginine, [^13^C_6_]‐lysine, or [^2^H_3_]‐leucine, or heavy water (^2^H_2_O).^17^, ^24^, ^28^, ^29^, ^30^ Figure 1 illustrates the general workflow of a protein turnover experiment, which typically consists of an experimental phase and an analysis phase. During the experimental phase, animals or cells are metabolically labeled with a chosen stable isotope to allow enrichment of label into newly synthesized proteins, and tissues are collected at several time points for later analysis. Metabolically labeling *C. elegans* and *D. melanogaster* can be achieved by providing ^15^N or via heavy amino acids by feeding of fully labeled *E. coli* or yeast, respectively.^17^, ^24^, ^31^, ^32^, ^33^, ^34^ Yeast labeling can be achieved by growing in media containing solely ^15^N or supplementing a heavy amino acid for which the yeast strain is auxotrophic.^17^, ^24^ Cells or animals will be kept on this synthetic diet until defined time points are reached and then they are harvested or sacrificed. MS data‐dependent acquisitions will be performed on several biological replicates of cells or tissue protein samples from each time point. Specialized downstream analysis is performed, for which several software tools are available, such as SILACtor,^35^ Topograph,^36^ ProTurn,^37^ DeuteRater,^38^ and others.^3^, ^4^, ^31^, ^39^For protein turnover analysis, the fraction of each protein that is newly synthesized over time is calculated and used to determine a final turnover rate or half‐life for each protein. Additional discussions and further details on labeling strategies, acquisition methods, and software analysis tools available for the determination of protein turnover rates using MS are available in other review articles.^17^, ^24^, ^25^

Calculating rates of protein synthesis and degradation is often more complicated than calculating turnover rates alone. Some of the traditional MS‐based methods rely on the assumption that proteins are at steady state at the time of labeling and there is no cellular proliferation, and therefore assume that turnover rates are equivalent to both synthesis and degradation rates. When protein abundances change and steady state does not apply, it is unclear whether the effects are due to changes in protein synthesis or degradation. These cases require additional information and effort to account for these factors, such as accurate accounting of cell proliferation rates, using multiple labels, or other considerations.^40^, ^41^, ^42^, ^43^, ^44^, ^45^ Some methods do not require additional effort but are restricted in their application, such as the ‘flooding dose’ approach, which is used to calculate protein synthesis rates but over a very acute (less than 30 minute) time period.^46^

A number of powerful non‐MS based approaches have also been employed in the determination of protein synthesis, degradation, and turnover rates. In particular, the tracking of fusion proteins with a fluorescent tag has been widely applied in cell culture models or *C. elegans* to determine protein half‐lives.^47^, ^48^, ^49^, ^50^, ^51^ These approaches have the advantage of spatial resolution that is not possible with MS‐based approaches, so that turnover of proteins in individual cells or even specific cellular compartments can be determined, and movement of proteins between compartments can be tracked. In live animals, chemical labeling approaches, such as “SNAP‐tag” pulse‐chase labeling, are available for in vivo imaging of proteins in live mice, followed by ex vivo determination of in vivo protein turnover rates.^52^ Aside from fluorescent and chemical labeling approaches, a method of directly assessing translational elongation rates in live animal in vivo has been very recently developed. This method applies the time‐resolved delivery of specific inhibitors of translational initiation and elongation in combination with polysome profiling to determine rates of translational elongation, both as tissue averages and at the level of individual transcripts.^53^

Alternative MS‐based workflows have also been employed to identify and quantify the relative abundances or PTM‐status of known, or presumed, LLPs. Protein aggregates and insoluble inclusions, for example, tend to be long‐lived because they evade protein turnover machinery. A number of methods exist to purify fractions of insoluble protein aggregates,^54^ or the insolublome,^55^, ^56^ to enrich for these presumably LLP fractions. Components of the ECM also tend to be long‐lived, and workflows to effectively prepare ECM protein fractions for MS have been developed.^57^ LLP populations also tend to be enriched with PTMs such as ubiquitination, oxidation, crosslinking, and carbamylation, which are sometimes used alone or in combination with other fractionation strategies to identify and quantify LLPs.^13^, ^58^, ^59^, ^60^

## 5 Protein Turnover Analysis During Aging and Age‐related Diseases and Possible Interventions

### 5.1 Protein Turnover in Invertebrate Aging and Longevity

As the changes in protein half‐lives and the decline in proteostasis are quite substantial during aging and age‐related diseases, increasing studies have emerged assessing global proteome turnover with aging as recently reviewed.^24^ Several studies have been performed in invertebrate model systems such as *C. elegans*, clearly characterizing the dependencies between protein turnover and aging. Comprehensive studies assessing proteostasis during *C. elegans* aging demonstrated a decrease in global proteome turnover in aged adult nematodes.^31^, ^32^, ^33^, ^34^ Interestingly, this decline is reversed in long‐lived strains, such as the insulin‐like growth factor receptor (daf‐2^–/–^) mutant.^31^, ^34^

Slower turnover rates have not only been reported with normal, healthy aging, but also with age‐related diseases in invertebrate models. For example, Visscher et al. investigated a *C. elegans* Parkinson's disease model that expresses α‐synuclein and found that the disease strain incorporates heavy lysine at a significantly slower rate in its proteome compared to the control strain^31^ and undergoes an accelerated decline of protein turnover rates compared with the normally aging strain. However, trends and changes of protein turnover with aging can certainly vary between different model systems, tissues, etc., and the protein turnover responses can be quite complex and heterogeneous. For example, a study by Dhondt et al.^32^ measured turnover slowdown during aging in *C. elegans*, and they discuss the heterogeneity of protein turnover patterns with age and show diverging turnover patterns. Interestingly, protein turnover of ubiquitin‐proteasome and antioxidant systems were described as well preserved over time, which could be a quality control mechanism and protective strategy in aging worms. Additional studies by Vukoti et al. also emphasize that turnover rates can change very differently for individual proteins—for example, they mention that new protein synthesis occurs in late stage *C. elegans*, and that the late‐life increase of newly synthesized protein was especially high for ribosomal proteins and ATP synthases.^61^ Such knowledge assessing protein turnover of individual proteins or different classes of proteins enables to better understand impact on cellular function or decline. Overall, protein turnover changes appear rather complicated, and measurements of protein turnover for individual proteins these using large scale proteomics methodologies described here, rather than applying bulk technologies, provide unique granular insights into dynamic turnover changes in cells.

Recently, protein turnover has been investigated in a series of different mutants, such as long‐lived or short‐lived strains with the goal to assess interventions in invertebrates to reverse aging phenotypes. For example, in the long‐lived insulin‐like growth factor (IGF‐1) receptor mutant (daf‐2^–/–^), most proteins (≈56%) showed longer half‐lives during development, exhibiting a slowdown in protein turnover, specifically for translation‐related and mitochondrial proteins.^62^ The authors concluded that lowering translational efficiency extended rather than shortened the lifespan in *C. elegans*, and that potentially the reduced insulin/IGF‐1 signaling may result in an energy‐conserving state, which may lead to improved protection of proteins. However, Visscher et al. showed that during adulthood (day 5) the opposite trend was seen, with long‐lived daf‐2 mutant worms having higher global protein turnover rates than strains with normal lifespan,^31^ despite having slower turnover during development. Further, the long‐lived strains have relatively preserved global protein turnover rates between development (day 2) and the first day of adulthood (day 5), while turnover rates decline during aging in strains with normal lifespan, and are further declined in short‐lived strains.^31^, ^34^ Collectively, these reports suggest that the maintenance of higher proteome turnover during adulthood may be important in determining lifespan in *C. elegans*. However, the molecular mechanisms underlying this relationship remain unclear.

### 5.2 Protein Turnover in Mammalian Aging and Longevity

Over the last two decades, in part due to advanced stable isotope labeling strategies and advancements in instrumentation technologies and software tools, in‐depth protein turnover aging studies have been performed in mammalian systems including in aging intervention studies, in vivo. The Rabinovitch group has comprehensively investigated protein turnover in various mouse studies either comparing young and old mice, using various models of healthy aging and longevity, including mice overexpressing mitochondria targeted catalase (mCAT),^28^ dietary restriction,^29^, ^63^ and rapamycin treatment.^29^, ^63^ Different tissues have been investigated, such as muscle,^64^ liver,^63^ or heart^29^ and the effects of caloric restriction as well as rapamycin as interventions for protein turnover have been assessed, as recently summarized.^24^ Thompson et al.^65^ also found a shift to dramatically slower protein turnover associated with long life when investigating long‐lived Pit‐1DW mice. The authors mention that reduced protein replacement rates were observed in these mouse models for hepatic proteins, and that these replacement rates could be directly correlated to maximum life span extension. The authors hypothesized that reduced hepatic protein replacement rates may evolve as a potential target for interventions that delay aging in mammals.^65^ In general, scientists have found that interventions that slow aging and extend lifespan in mammals also globally reduce rates of proteome turnover^28^, ^29^, ^40^, ^63^, ^65^ and improve protein quality in mouse tissues, suggesting that preservation of protein quality and stability may be a common component of aging interventions and longevity. Intriguingly, the association of slower protein turnover (and improved protein quality) with longevity also extends to rodent species with diverse lifespans. A recent SILAC study by Swovick et al. examined global protein turnover rates in cultured fibroblasts from eight rodent species with diverse lifespans—including mouse, rat, hamster, guinea pig, beaver, chinchilla, blind mole rate, and naked mole rat—found that global protein turnover rates negatively correlated with the lifespans of the species.^66^ However, while slower proteome turnover is unambiguously associated with longer lifespan in mammalian studies conducted thus far, a largely opposite trend has been reported invertebrate models, such as *C. elegans*,^31^, ^34^, ^62^ where preservation of faster (youthful) rates of protein turnover appear to be beneficial in old age. The reason for the discrepancy between rodent and invertebrate models remains unclear but we speculate that the primary cellular mechanisms driving aging in *C. elegans* versus rodents is different. For example, *C. elegans* aging is marked by severe proteome imbalance and protein aggregation and is mitigated by mutations or downregulation of specific proteins, which overall decreases protein aggregation.^67^, ^68^, ^69^ Therefore, the need for higher rates of proteostasis may supersede the potential benefits of maintaining higher quality and longer‐lived proteins in this model. In addition, expression of genes encoding insoluble proteins observed in aged nematodes was knocked down using RNAi, and effects on lifespan were measured; indeed, 41% of genes tested were shown to extend lifespan after RNAi treatment.^69^ There was consensus in the hypothesis that widespread protein insolubility and aggregation is an inherent part of aging, and that it may influence both lifespan and neurodegenerative disease.^67^, ^69^

It should be noted that in multicellular organisms and intact animals protein turnover rates for the same protein in the same organism at a given time point are typically quite different in different tissues, for example, in muscle, turnover rates tend to be slow, while in liver, turnover rates are typically rather fast.^24^ Hammond et al. performed a detailed study assessing these tissue‐specific effects for 1088 proteins in a rodent animal model (bank vole, *Myodes glareolus*).^70^ Comparative analysis of the four tissues revealed different median rates of degradation (kidney: 0.099 d^−1^; liver 0.136 d^−1^; heart, 0.054 d^−1^, and skeletal muscle, 0.035 d^−1^).^70^ Clearly, tissue specific differences and protein turnover changes with aging will be very interesting, and initial studies have provided some insights into differential regulation of protein turnover. Further studies comparing the changes in turnover in various tissues during both normal aging and in longevity models will be needed to determine whether aging and aging‐interventions drive tissue‐specific changes in protein turnover.

Assessing proteome turnover in mice during aging also revealed interesting and surprising insights into electron transport chain complexes (ETC), that uniquely are large assemblies of ETC protein subunits. Respiratory chain protein turnover rates in mice are highly heterogeneous between subunits of ETCs but strikingly conserved across tissues, ages, and treatments.^30^, ^64^ Mitochondrial protein homeostasis is of key relevance for preventing mitochondrial dysfunction, and tissue‐specific changes in protein turnover play an important role in muscle function.^64^ In general, muscle function and muscle aging seem to be directly linked to dynamic changes in protein turnover as recently presented in a study of the mouse skeletal muscle proteome during denervation‐induced atrophy.^71^ Denervation caused a rapid loss of muscle mass by reducing protein synthesis and enhancement of protein breakdown.

The role of protein turnover can be many‐fold and highly relevant for diverse cellular processes. For example, Schroeter et al. determined the regulation of protein turnover during differentiation of podocytes, specialized filtration cells of the kidney.^72^ While undifferentiated podocytes showed high expression of proteasomal proteins, differentiated podocytes showed high expression of lysosomal proteins. A global increase in stability of proteins was observed in differentiated podocytes, and mitochondrial, cytoskeletal, and membrane proteins were stabilized. Interestingly, changes in protein half‐lives strongly contributed to changes in protein abundances. The authors hypothesized that these regulatory mechanisms may be important in conditions of increased podocyte stress and damage.

Protein turnover also plays a role in the management of cellular resources in response to environmental conditions. For example, large changes in protein turnover are observed during different conditions of low nutrient signaling, and caloric restriction appears to reduce rates of protein synthesis and degradation. As Mathis et al. demonstrated, dietary changes and signaling can impact assembly of new ribosomes, component exchange, and ribosomal repair,^73^ as well as increased levels of autophagy to recycle cellular proteins into basic components for re‐use. The ability of nutrient‐responsive pathways such as mTOR to fine‐tune cellular proteostasis machinery in this way is indeed one reason they are attractive targets for therapeutics to improve lifespan and health span.

Finally, defects or changes in proteostasis may be deeply involved with conditions of accelerated aging. A study in Hutchinson‐Gilford progeria syndrome (HGPS), a disease manifested by accelerated aging and premature death (usually in the teens), revealed a significant increase in protein turnover and an elevation in protein synthesis.^74^ This was caused by enhanced ribosome biogenesis and nucleolar expansion. The authors noted that lamin A depletion during progeria drives nucleolar expansion establishing a connection between the nuclear lamina, nucleolar organization, and regulation of protein synthesis output. This is particularly interesting as the study shows that this increased ribosome biogenesis and activity appears to be a key driver of premature aging in HGPS, also suggesting that limiting ribosome biogenesis may extend lifespan.

### 5.3 Age‐Related Protein Turnover in Humans

Several studies have been performed in humans examining protein turnover in sarcopenia or age‐related skeletal muscle atrophy. Muscle strength and mass is typically maintained until middle age, after which accelerated losses occur in both.^75^ Isotopic labeling studies in humans are not trivial to perform: some studies have continuously infused l‐[1‐^13^C]‐leucine and subsequently collected skeletal muscle biopsies. It was found that aging reduced the protein synthesis rates in the skeletal muscle, which may be relevant or even causal during age‐related decline in muscle mass.^18^, ^22^ Both resistance and aerobic exercise training appear to increase muscle protein synthesis and improve muscle function that may ameliorate aging effects.^18^, ^76^

For human in vivo studies, there are clearly limitations as far as changes in diet that would require stable‐isotope supplements. Amino acid infusion studies in humans are largely limited as a continuous infusion can only be feasibly conducted over the course of a few hours. Thus, in vivo human studies are largely limited to measuring turnover of short‐lived proteins, which incorporate detectable amounts of heavy isotope over the length of the study. However, this is not a limitation in human cell line and tissue culture studies.^77^ Notably, one study overcame of limitations of traditional in vivo turnover studies in humans by examining ^14^C incorporated into proteins as a result of the “bomb pulse,” or the sudden increase of carbon‐14 in the Earth's atmosphere between 1950 and 1963 as a result of nuclear bomb tests.^78^ By measuring ^14^C/^12^C ratios in lens proteins, the authors estimated turnover of very long lived crystallin proteins of the lens, observing a low‐level turnover in soluble crystallins, and no turnover among insoluble crystallins (^14^C/^12^C ratios consistent with the age of the cells).^78^

## 6 Concluding Remarks

In general, proteomics studies may comprise much more than just measuring differential protein expression. Particularly when combining multiple different types of protein measurements, a systems biology perspective can help to better and more comprehensively understand the biological complexity involved with aging and age‐related diseases. While further studies are still needed and any protein marker candidate resulting from a protein turnover study will still need in‐depth validation, MS‐based protein turnover studies allow to investigate a scientific space of proteomics that is often underexplored. However, gaining insights into dynamic PTM profile changes, potential protein aggregation mechanisms, and foremost investigating protein turnover and changes in proteostasis and protein half‐lives will be crucial during future aging studies. Mass spectrometric workflows have already elevated protein turnover studies to a level of unprecedented detail, demonstrating the dynamic changes of protein half‐lives for individual proteins and entire proteomes. Since the past few years, these approaches have been applied to the study of aging and longevity, researchers have uncovered that the turnover of specific proteins and pathways are impacted more strongly by aging and aging interventions than others. A striking correlation observed between reduced protein turnover and slower aging has led to new hypotheses suggesting that targeting the proteostasis machinery to slow down protein turnover may be a promising approach to mitigate age‐related diseases.

## Conflict of Interest

The authors declare no conflict of interest.
